# *Pseudomonas cannabina* pv. *alisalensis* Virulence Factors Are Involved in Resistance to Plant-Derived Antimicrobials during Infection

**DOI:** 10.3390/plants11131742

**Published:** 2022-06-30

**Authors:** Nanami Sakata, Takumi Haraguchi, Shunsuke Masuo, Takako Ishiga, Yasuhiro Ishiga

**Affiliations:** 1 Faculty of Life and Environmental Sciences, University of Tsukuba, 1-1-1 Tennodai, Tsukuba 305-8572, Ibaraki, Japan; sakata.nanami.td@alumni.tsukuba.ac.jp (N.S.); takku_yakult@icloud.com (T.H.); masuo.shunsuke.fp@u.tsukuba.ac.jp (S.M.); takakoishiga@gmail.com (T.I.); 2 Microbiology Research Center for Sustainability (MiCS), University of Tsukuba, 1-1-1 Tennodai, Tsukuba 305-8572, Ibaraki, Japan

**Keywords:** *Pseudomonas cannabina* pv. *alisalensis*, resistance-nodulation-cell division transporter, type-three secretion system, phytoalexin, brassinin, glucosinolate, cabbage

## Abstract

Bacteria are exposed to and tolerate diverse and potentially toxic compounds in the natural environment. While efflux transporters are generally thought to involve bacterial antibiotic resistance in vitro, their contributions to plant bacterial virulence have so far been poorly understood. *Pseudomonas cannabina* pv. *alisalensis* (*Pcal*) is a causal agent of bacterial blight of Brassicaceae. We here demonstrated that NU19, which is mutated in the resistance-nodulation-cell division (RND) transporter encoded gene, showed reduced virulence on cabbage compared to WT, indicating that the RND transporter contributes to *Pcal* virulence on cabbage. We also demonstrated that brassinin biosynthesis was induced after *Pcal* infection. Additionally, the RND transporter was involved in resistance to plant-derived antimicrobials and antibiotics, including the cabbage phytoalexin brassinin. These results suggest that the RND transporter extrudes plant-derived antimicrobials and contributes to *Pcal* virulence. We also found that the RND transporter contributes to *Pcal* virulence on Brassicaceae and tomato, but not on oat. These results suggest that the RND transporter contributes to *Pcal* virulence differentially depending on the host-plant species. Lastly, our expression-profile analysis indicated that the type-three secretion system (TTSS), which is essential for pathogenesis, is also involved in suppressing brassinin biosynthesis. Taken together, our results suggest that several *Pcal* virulence factors are involved in resistance to plant-derived antimicrobials and bacterial survival during infection.

## 1. Introduction

Plants produce diverse specialized secondary metabolites to protect against pathogens and pests [1]. Specialized metabolites differ between plant clades [2]. The simplest functional definitions recognize “phytoalexins” as metabolites that are synthesized de novo in response to a pathogen, and “phytoanticipins” as constitutively biosynthesized infection inhibitors [3]. Phytoanticipins and phytoalexins are structurally diverse and different in plant species. So far, at least 44 phytoalexins have been isolated from Brassicaceae, most of which are derived from the amino acid tryptophan [4]. These defense metabolites have inhibitory activity in vitro against various bacteria and fungi, and they confer disease resistance in plant–pathogen interactions [4,5,6,7].

The Brassicaceae family includes many economically important crops. More than 40 phytoalexins have been identified from cultivated and wild Brassicaceae. *Brassica* species produce indole sulfur phytoalexins, which are hallmarks of the Brassicaceae with different subsets produced by different edible crucifers [8,9]. The role of phytoalexins in pathogen resistance has been well-studied in the model plant *Arabidopsis thaliana*. Camalexin is a major phytoalexin of *A. thaliana*, and its production can be induced in *A. thaliana* leaves by a range of biotrophic and necrotrophic plant pathogens [4,10]. Camalexin antimicrobial activity was shown in vitro against bacteria, oomycetes, and fungi [11,12,13,14,15,16,17]. A mutation in the *PHYTOALEXIN DEFICIENT 3* (*PAD3*) gene abolishes camalexin biosynthesis, resulting in enhanced susceptibility to necrotrophic pathogens, including *Botrytis cinerea* [16,18] and *Alternaria brassicicola* [19,20]. Several studies highlighted the importance of camalexin in response to hemibiotrophic pathogens [21,22], although camalexin accumulation was not always correlated with pathogen resistance. For instance, camalexin production was induced in response to various *Pseudomonas syringae* strains, but a *pad3* mutant showed the same susceptibility to those strains [23,24,25].

Klein and Sattely (2017) identified the biosynthetic genes required to generate the cruciferous phytoalexin brassinin. Brassinin is a glucosinolate downstream product and is a starting point for the various other phytoalexins [26]. Brassinin is not present in *A. thaliana* but is produced by many cultivated Brassica species. The inability of *A. thaliana* to synthesize or tailor brassinin is associated with the absence of enzymes, including brassinin-associated β-glucosidase (BABG) and dithiocarvamate 5-methyltransferase (DTCMT) [8]. Brassinin antifungal activity in vitro has been reported [13]. Brassinin primarily targets mitochondrial functions in *A. brassicicola*, then induces secondary effects such as reactive oxygen species (ROS) production and changes in lipid homeostasis [27]. Camalexin contributes to plant resistance against various fungal and oomycete pathogens [11,18,21,28,29]. However, few studies have investigated the importance of brassinin in plant resistance, and especially focused on the importance of phytoalexin in resistance against bacterial pathogens.

*Pseudomonas cannabina* pv. *alisalensis* (*Pcal*) is a causal agent of bacterial blight of Brassicaceae [30]. *Pcal* has a wide plant-host range: the Brassicaceae family (including cabbage, broccoli, Japanese radish, Chinese cabbage), tomato, and portions of Poaceae families such as oat (*Avena strigosa*) and timothy (*Phleum pratense*) [30]. Currently, copper fungicides and antibiotics have mainly been used for bacterial disease control. However, bacterial strains (including a *Pcal* strain) have developed a resistance against these chemicals [31]. To develop new strategies for *Pcal* disease control, we need to identify *Pcal* infection mechanisms. We previously identified potential *Pcal* virulence factors [32]. Multiple virulence factors are needed for successful infection such as the type-three secretion system (TTSS), membrane transporters, transcriptional factors, and amino-acid metabolism [32]. Among these mutants, a NU19 mutant (where Tn*5* is inserted in the resistance-nodulation-cell division (RND) transporter encoded gene (PMA4326_12408)), showed reduced virulence on cabbage [32]. However, the function of the RND transporter in *Pcal* virulence remains largely unclear.

For successful infection, plant pathogens need to eliminate the effects of host-derived antimicrobial compounds through extruding antimicrobials outside the cell, suppressing biosynthesis, and converting them to ineffective ones [7,33]. To extrude antimicrobials, bacteria have five structural groups of multidrug resistance (MDR) efflux-pump transporters: RND, small multidrug resistance, multiantimicrobial extrusion, the major facilitator superfamily, and ATP-binding cassette superfamilies. The RND efflux system functions to extrude various substrates, including antibiotics and host-derived molecules [34]. Fan et al. (2011) demonstrated that the sax (*survival in Arabidopsis extracts*) genes in *P. syringae pv. tomato* (*Pto*) DC3000 are required to overwhelm isothiocyanate-based defenses and facilitate a disease outcome. The *sax* genes form a subgroup of the RND efflux system [7]. In *P. syringae*, there are different operons for the RND efflux-pump transporter, *mexAB-oprM* and *mexEF-oprN*. *mexAB-oprM* deletion mutants in *Pto* DC3000, *P. syringae* pv. *phaseolicola* (*Pph*) 1448A, *P. syringae* pv. *syringae* (*Psy*) B728a, and *P. amygdali* pv. *tabaci* (formerly *P. syringae* pv. *tabaci*; *Pta*) 6605 exhibited increased antimicrobial susceptibility [35,36,37]. Helmann et al. (2022) demonstrated that *Psy* B728a MexB contributes to virulence differentially depending on the host-plant species. Therefore, although it is tempting to speculate that the *Pcal* RND transporter also contributes to virulence differentially depending on the host-plant species, few RND transporter studies focused on host-derived phytoalexin and its virulence contributions on different host plants.

We here investigated the importance of the RND transporter in *Pcal* virulence by inoculating the NU19 strain [32] on Brassicaceae crops. We demonstrated that brassinin accumulated in several brassica crops, and is induced by *Pcal* infection. We also showed that the RND transporter is involved in diverse antimicrobial sensitivity, including brassinin. Moreover, our results also indicated that the TTSS might be involved in suppressing brassinin biosynthesis. Together, our results suggest that several *Pcal* virulence factors are involved in resistance to plant-derived antimicrobials and bacterial survival during infection.

## 2. Results

### 2.1. RND Transporter Contributes to Pcal Virulence

**Figure 1:**
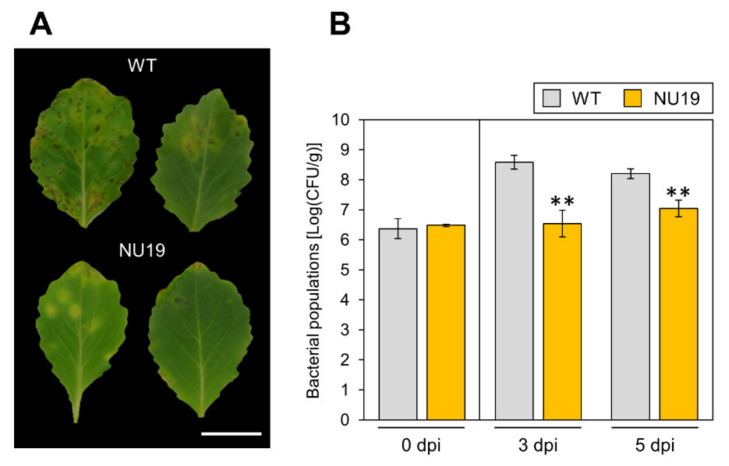
Disease symptoms (**A**) and bacterial populations (**B**) on cabbage leaves dip-inoculated with *Pseudomonas cannabina* pv. *alisalensis* KB211 WT and NU19. Cabbage plants were dip-inoculated with 5 × 10 ^7^ CFU/mL of inoculum containing 0.025% Silwet L-77. The bacterial populations in the plant were evaluated at 0, 3, and 5 dpi. The leaves were photographed at 5 dpi. Scale bar shows 2 cm. Vertical bars indicate the standard error for at least three independent experiments. Asterisks indicate a significant difference from the *Pcal* WT in a *t*-test (** *p* < 0.01).

To investigate the RND transporter contributions to *Pcal* virulence, we conducted an inoculation assay with the RND transporter mutant NU19, which was isolated as a reduced virulence strain in a previous screening [32]. We firstly confirmed no significant differences in bacterial growth in KB medium between WT and NU19 after 12 h and 24 h incubation (Supplementary Figure S1). When we dip-inoculated plants with WT, cabbage showed chlorosis and necrosis (Figure 1A). However, cabbage inoculated with NU19 showed reduced symptoms (Figure 1A). Bacterial populations were also significantly reduced in plants inoculated with NU19 (Figure 1B). These results indicate that the RND transporter contributes to *Pcal* virulence on cabbage.

### 2.2. Brassinin Biosynthesis Is Induced after Pcal Infection

**Figure 2:**
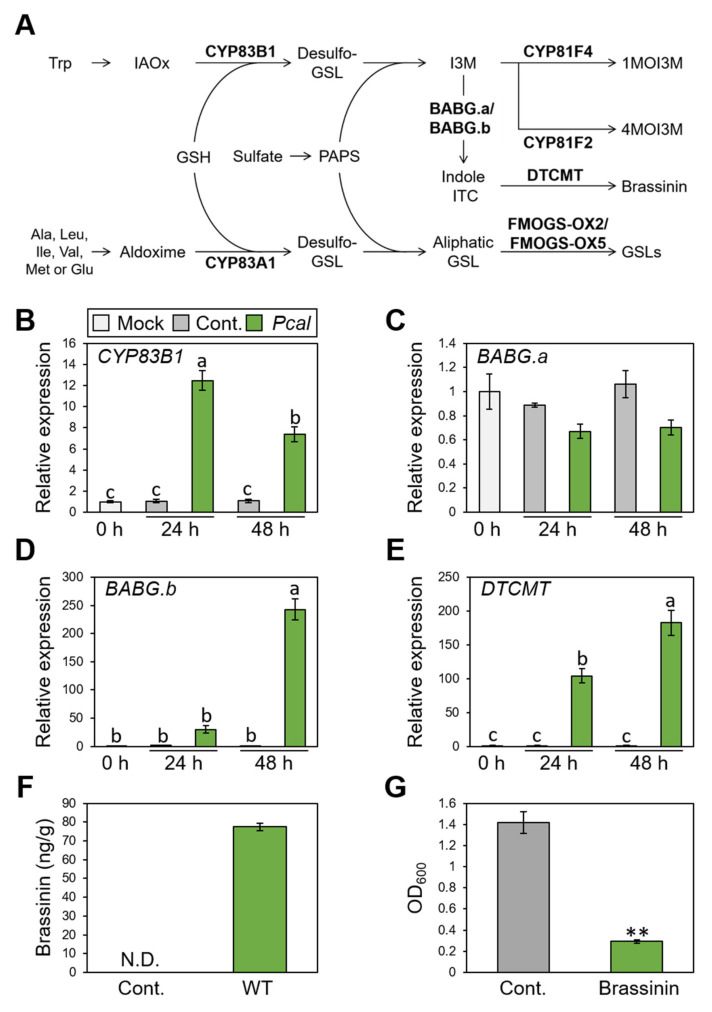
Expression profiles of brassinin-related genes and brassinin production during *Pseudomonas cannabina* pv. *alisalensis* KB211 WT infection, and antimicrobial activity of brassinin. (**A**) The aliphatic and indolic glucosinolate biosynthesis pathways in cabbage. Schemic biosynthetic pathways with the specific biosynthetic enzyme locations used in this study are shown in bold. GSH, glutathione; GSL, glucosinolate; IAOx, indole-3-acetaldoxime; ITC, isothiocyanate; I3M, indole glucosinolates; PAPS, 3′-phosphoadenosine-5′-phosphosulfate; 1MOI3M, 1-Methoxyindole-3-yl methyl glucosinolate; 4MOI3M, 4-Methoxyindole-3-yl methyl glucosinolate. Brassinin biosynthesis gene expression profiles after syringe-inoculation with water (mock), or *Pseudomonas cannabina* pv. *alisalensis* KB211 WT. Expression profiles of *CYP83B1* (**B**), *BABG.a* (**C**), *BABG.b* (**D**), and *DTCMT* (**E**) were determined 24 and 48 h after inoculation with 5 × 10^5^ CFU/mL of WT or mock water-inoculated control, using real-time quantitative reverse-transcription PCR with gene-specific primer sets. Expression in cabbage was normalized using *BoUBQ1*. Vertical bars indicate the standard error for three biological replicates. Different letters indicate a significant difference among treatments based on a Tukey’s honestly significant different test (*p* < 0.05). (**F**) Total brassinin production in cabbage after syringe inoculation with *Pcal* WT or with water as a control. Cabbage leaves were collected at 48 hpi and were extracted with 80% methanol. Then, total brassinin were quantified by RP-LC-ESI-MS/MS. Vertical bars indicate the standard error for at least three independent experiments. N.D. indicates not detected. (**G**) Bacterial growth in LB medium after 24 h incubation with or without brassinin. The bacterial suspension was standardized to an OD_600_ of 0.01 in LB and coincubated with or without 200 µM brassinin. After 24 h, bacterial growth was measured at OD_600_. Asterisks indicate a significant difference from the water-treatment control in a *t*-test (** *p* < 0.01).

The RND transporter functions to extrude a wide range of substrates, including antibiotics and host-derived molecules [34]. We then hypothesized that NU19 exhibited reduced virulence by impairment in cabbage-derived antimicrobial efflux. Therefore, we firstly examined whether cabbage secondary metabolites, glucosinolate biosynthesis, are induced after *Pcal* infection. We investigated expression profiles of brassinin biosynthesis-related genes (*CYP83B1*, *BABG.a*, *BABG.b*, and *DTCMT*), indole glucosinolate biosynthesis-related genes (*CYP81F2* and *CYP81F4*), and aliphatic glucosinolates biosynthesis-related genes (*CYP83A1*, *FMOGS-OX2*, and *FMOG-OX5*) (Figure 2A). Brassinin biosynthesis-related genes, except *BABG.a*, showed greater expression after *Pcal* infection (Figure 2B–E). However, aliphatic glucosinolate biosynthesis-related genes, including *CYP83A1*, *FMOGS-OX2*, and *FMOGS-OX5*, showed less expression after infection (Supplementary Figure S2A–C). Moreover, the indole glucosinolate pathway, *CYP81F2* and *CYP81F4*, also showed greater expression after infection, same as the brassinin biosynthesis pathways (Supplementary Figure S2D,E).

We next examined brassinin quantification using LC-MS/MS. The brassinin amount reached around 80 ng/g after *Pcal* infection at 48 h post inoculation (hpi) (Figure 2F). Moreover, brassinin has antimicrobial activity against *Pcal* (Figure 2G). Taken together, these results indicate that brassinin functions as a phytoalexin against *Pcal* infection.

### 2.3. RND Transporter Contributes to Resistance to Diverse Toxicants

**Figure 3:**
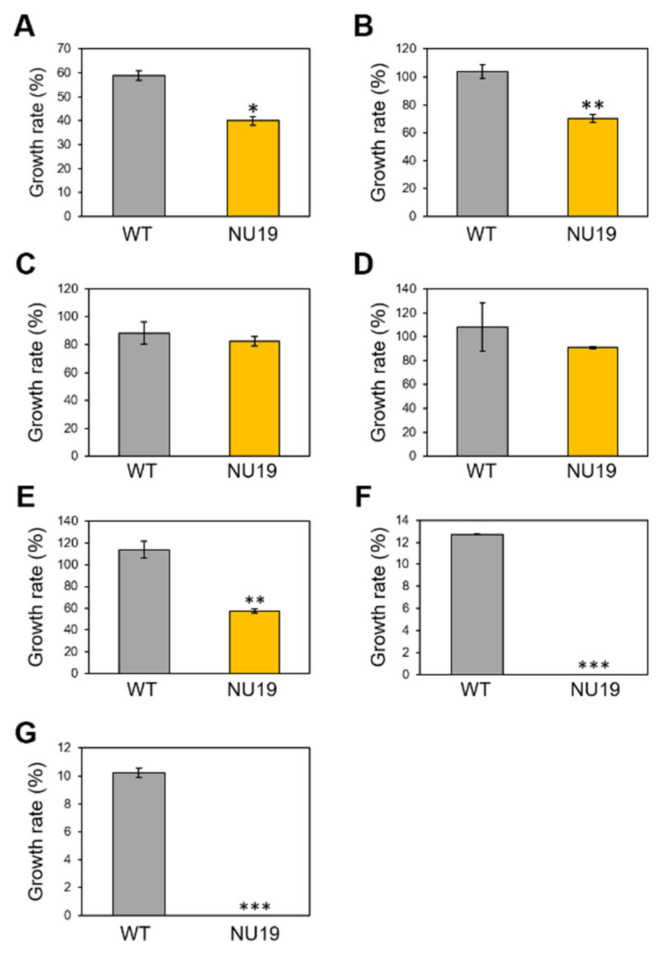
Growth rate of *Pseudomonas cannabina* pv. *alisalensis* KB211 WT and NU19 in KB medium with or without plant-derived antimicrobials. The bacterial suspensions were standardized to an OD_600_ of 0.01 with KB, and after 6 h incubation, 200 µM brassinin (**A**), sulforaphane (**B**), camalexin (**C**), daidzein (**D**), genistein (**E**), indole (**F**), and phloretin (**G**) were added to each sample. Bacterial growth was measured at OD_600_ after 24 h incubation. Asterisks indicate a significant difference from the *Pcal* WT in a *t*-test (* *p* < 0.05, ** *p* < 0.01, *** *p* < 0.001).

To investigate whether the RND transporter is involved in resistance to various toxicants, we firstly examined the brassinin sensitivity of WT and NU19. The NU19 growth rate was reduced compared to WT (Figure 3A), suggesting that the RND transporter contributes to brassinin resistance. We next examined the sensitivity to other plant-derived metabolites. NU19 was significantly susceptible to sulforaphane, genistein, indole, and phloretin in these experimental conditions (Figure 3B–G). Furthermore, NU19 was more sensitive to spectinomycin and streptomycin than WT (Table 1). Taken together, these results indicate that the RND transporter contributes to resistance to several plant-derived antimicrobials and antibiotics.

### 2.4. RND Transporter Contributes to Pcal Virulence on Multiple Host Plants

**Figure 4:**
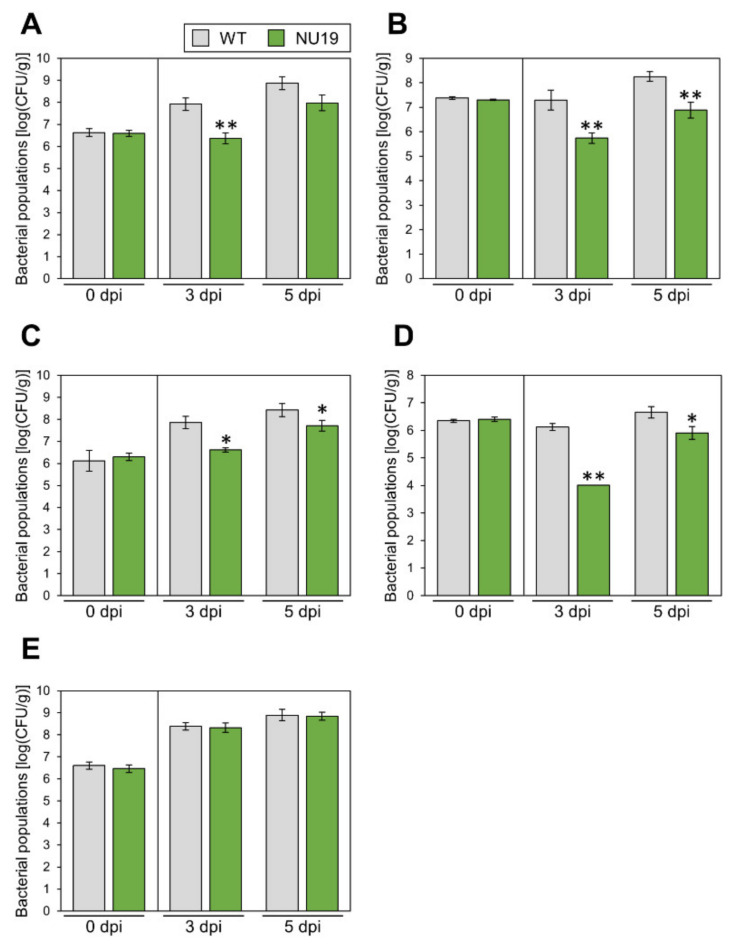
Bacterial populations of *Pseudomonas cannabina* pv. *alisalensis* KB211 WT and NU19 in brocolli (**A**), Japanese radish (**B**), Chinese cabbage (**C**), tomato (**D**), and oat (**E**). All plants were dip-inoculated with 5 × 10^7^ CFU/mL of inoculum containing 0.025% Silwet L-77. The bacterial populations in the plant were evaluated at 0, 3, and 5 dpi. Vertical bars indicate the standard error for at least three independent experiments. Asterisks indicate a significant difference from the *Pcal* WT in a *t*-test (* *p* < 0.05, ** *p* < 0.01).

We next investigated whether the RND transporter contributes to *Pcal* virulence on multiple host plants. Disease symptoms and bacterial populations of NU19 were reduced in Brassica plants, including broccoli, Japanese radish, and Chinese cabbage (Figure 4A–C; Supplementary Figure S3A–C). These results indicate the RND transporter contributes to *Pcal* virulence on Brassica crops. Moreover, disease symptoms and bacterial populations of NU19 were reduced compared to WT in tomato (Figure 4D; Supplementary Figure S3D). Interestingly, however, disease symptoms and bacterial populations of NU19 and WT were almost the same in oat (Figure 4E; Supplementary Figure S4E). These results suggest that the RND transporter contributes less or does not contribute to disease on oat. Taken together, although the RND transporter contribution to *Pcal* virulence differed in infection on oat, the RND transporter contributes to disease on multiple host plants.

### 2.5. TTSS Suppresses Brassinin Biosynthesis

**Figure 5:**
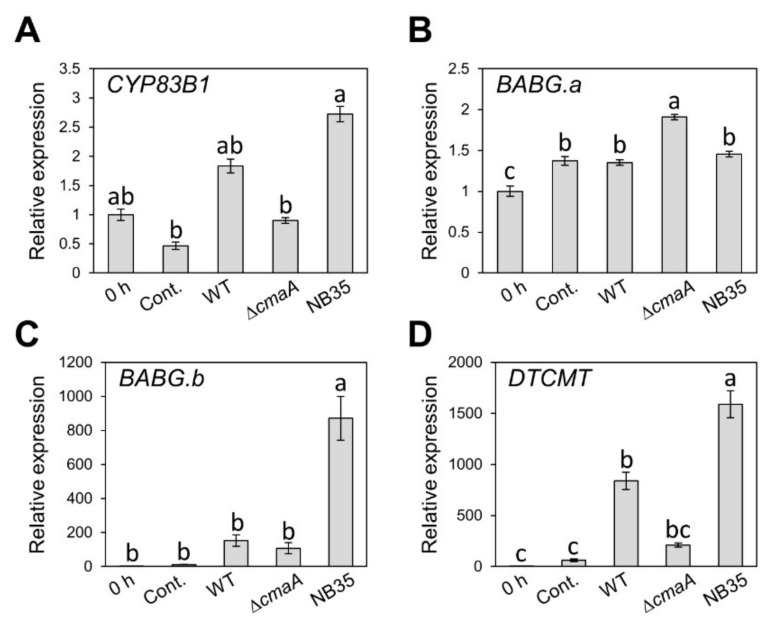
Gene expression profiles of brassinin biosynthesis-related genes after syringe inoculation with water (mock), or *Pseudomonas cannabina* pv. *alisalensis* KB211 WT, Δ*cmaA*, and NB35 (TTSS mutant). Expression profiles of *CYP83B1* (**A**), *BABG.a* (**B**), *BABG.b* (**C**), and *DTCMT* (**D**) were determined 6 h after inoculation with 5 × 10^7^ CFU/mL of WT, Δ*cmaA*, NB35 or mock water-inoculated control, using real-time quantitative reverse transcription PCR with gene-specific primer sets. Expression in cabbage was normalized using *BoUBQ1*. Vertical bars indicate the standard error for three biological replicates. Different letters indicate a significant difference among treatments based on a Tukey’s honestly significant different test (*p* < 0.05).

We demonstrated that brassinin biosynthesis is induced by *Pcal* infection. Therefore, it is tempting to speculate that efflux or detoxification of host-derived antimicrobials, including brassinin, is a critical step for successful *Pcal* infection. Thus, we assumed that the RND transporter is not the only virulence factor that suppresses brassinin accumulation. Therefore, to investigate whether the TTSS and coronatine (COR), which are important *Pcal* virulence factors [32,38], are involved in brassinin suppression, we examined the expression profiles of brassinin biosynthesis related-genes during infection with WT, NB35 (a TTSS mutant), and Δ*cmaA*. Since NB35 and Δ*cmaA* showed significantly reduced populations compared to WT [32,38,39], we first determined the time point at which these three strains have the same bacterial populations. Bacterial populations in cabbage inoculated with these strains were not significantly different at 6 hpi (Supplementary Figure S4). Therefore, we examined the expression profiles in plants inoculated with these *Pcal* strains at 6 hpi. All genes involved in brassinin biosynthesis, except *BABG.a*, showed significantly or tended to show greater expression during NB35 infection compared to WT and Δ*cmaA* (Figure 5A–D). These results indicate that the TTSS might be involved in brassinin biosynthesis suppression.

## 3. Discussion

Bacteria are exposed to, and tolerate, diverse and potentially toxic compounds in the natural environment [40]. While efflux transporters are generally thought to involve bacterial antibiotic resistance in vitro, their contributions to plant bacterial virulence have so far been poorly understood. We here demonstrated that NU19, which is mutated in the RND transporter encoded gene, showed reduced virulence on cabbage compared to WT (Figure 1), indicating that the RND transporter contributes to *Pcal* virulence on cabbage. We also demonstrated that brassinin biosynthesis was induced after *Pcal* infection (Figure 2). Additionally, the RND transporter was involved in resistance to several plant-derived antimicrobials and antibiotics, including brassinin (Figure 3). These results suggests that the RND transporter contributes to *Pcal* virulence through extruding host-derived antimicrobials. The RND transporter also contributes to *Pcal* virulence on Brassicaceae plants and tomato, but not on oat (Figure 4), suggesting that RND transporter contributes to *Pcal* virulence differentially depending on the host plant species. Lastly, our expression-profile analysis indicates that the TTSS is also involved in brassinin biosynthesis suppression (Figure 5). Taken together, our results suggest that several *Pcal* virulence factors are involved in resistance to plant-derived antimicrobials and bacterial survival during infection.

Brassinin biosynthesis was induced in response to *Pcal* infection (Figure 2). Moreover, indole glucosinolates biosynthesis-related gene expression, including brassinin, were induced after *Pcal* infection (Figure 2; Supplementary Figure S2D,E). Conversely, aliphatic glucosinolate biosynthesis-related gene expression was downregulated in response to *Pcal* infection (Supplementary Figure S2A–C). Indeed, in *A. thaliana*, when the aliphatic glucosinolate pathway is blocked because of a *cyp83a1* mutation, the pathways for indole glucosinolate and camalexin were enhanced [41]. One possible explanation for how the glucosinoalte synthetase *CYP83A1* gene mutation affects camalexin accumulation is that it may cause crosstalk between the aliphatic glucosinolates and indole glucosinolates biosynthetic pathways [41]. Consistent with this, our data also indicated that indole glucosinolate pathways, including brassinin, were induced after *Pcal* infection, while alipathic glucosinolate pathways were downregulated (Figure 2; Supplementary Figure S2). Accumulation of indole indolic metabolites has been observed in response to *P. syringae* [23,24,42,43], and these indole metabolites, such as camalexin, 4-methoxyglucobrassicin, and 4-hydroxyindole-3-carbonyl nitrile, are important in *A. thaliana* basal defense [19,43,44,45,46]. Importantly, the increased biosynthesis of various glucosinolate classes depends on the type of challenging pathogens [26]. Together, the rapid and precise regulation of glucosinolate biosynthesis work in response to pathogen infection, and the downstream products of indole glucosinolates might function as the cabbage defense metabolites against bacterial pathogens.

The RND transporter contributes to *Pcal* virulence on Brassicaceae crops (Figure 1 and Figure 4A–C). We also demonstrated that the RND transporter contributes to susceptibility to the cabbage phytoalexin brassinin (Figure 3A), suggesting that RND transporters contribute to *Pcal* virulence by providing resistance to host-derived antimicrobials. The *mexAB*-*oprM* deletion mutants of *Pto* DC3000, *Pph* 1448A, *Psy* B728a, and *Pta* 6605 exhibited increased susceptibility to antimicrobials, and reduced disease-symptom development and bacterial populations [35,36,37]. In *Pta*, the RND transporter contributed not only directly to extrude antimicrobials, but also indirectly to regulate motility and N-acyl-homoserine lactone (AHL) production [36]. Thus, further investigation will lead to understanding the importance of the RND transporter in bacterial virulence.

Brassinin biosynthesis-related genes showed greater expression during the TTSS mutant infection compared to WT and the COR mutant (Figure 5), suggesting that the TTSS is involved in suppressing brassinin biosynthesis. Bais et al. (2005) also demonstrated that the TTSS and perhaps other virulence factors under HrpL control (but not COR) are required for blocking the synthesis or exudation of antimicrobial compounds in *Pto* DC3000 [47]. Moreover, *P. syringae* HopZ1 targeted a host enzyme to suppress isoflavone biosynthesis in soybean, which are important secondary metabolites during plant–microbe interactions in soybean [48]. These results suggest that the TTSS suppressed host-derived antimicrobial biosynthesis in addition to those emitted by the RND transporter. Conversely, a phytoanticipin, sulforaphane, inhibits *P. syringae* TTSS genes [49]. Chemoproteomics analyses showed that sulforaphane covalently modified the cysteine at position 209 of HrpS, a key transcriptional factor controlling TTSS gene expression [49]. This study indicated that sulforaphane inhibited virulence gene expression instead of targeting general bacterial activity. Taken together, although further analysis will be needed, there is a possibility that plant-derived antimicrobials and bacterial virulence factors target each other.

Moreover, although the RND transporter contributes to *Pcal* virulence on multiple host plants (Figure 4A–D), the NU19 multiplication defect was not observed on oat, indicating that the RND transporter has less or no contribution to disease on oat (Figure 4E). *Psy* B728a MexB contributes to virulence in common bean, but was not required for growth in lima bean, fava bean, pepper, *Nicotiana benthamiana*, sunflower, and tomato [50]. Additionally, *Sclerotinia scleotiorum* induced both camalexin and aliphatic glucosinolate biosynthesis genes, while *B. cinerea* did not induce aliphatic glucosinolate and induced camalexin biosynthesis genes [14]. Therefore, the plant–microbe interaction must be considered. Moreover, despite the high degree of primary homology between two RND transporters, AcrAB-TolG and MexAB-OprM, these pumps do not efflux all substrates with equal efficiency [51]. These results indicated that RND transporters have substrate specificity. Related plant families generally make use of related chemical structures for defense [2]. Indeed, oat major specialized metabolites are amphiphilic saponins [52], while brassicaceae major specialized metabolites are glucosinolates and indole alkanoids [53]. Given these very different polarities, it is unlikely that the same RND transporter would be suitable to efflux them out of the cells. However, the reason for the difference in RND transporter contribution depending on the host plant remains unclear. Further studies on oat secondary metabolites and RND transporter roles in *Pcal* virulence on different host plants are necessary.

We here demonstrated that the RND transporter contributes to *Pcal* virulence on Brassicaceae plants. The RND transporter plays an important role in resistance to plant-derived antimicrobials and antibiotics. Moreover, we revealed that the TTSS might be involved in suppressing brassinin biosynthesis. Our study shed light on the importance of efflux or suppressing host-derived antimicrobials for successful bacterial infection. Further study on plant–bacterial interactions over host-derived metabolites will be needed to understand *Pcal* host diversity and virulence mechanisms.

## 4. Materials and Methods

### 4.1. Bacterial Strains, Plasmids, and Growth Conditions

The bacterial strains and plasmids used in this study are described in Supplementary Table S1. *Pseudomonas cannabina* pv. *alisalensis* strain KB211 (*Pcal* KB211) was used as the pathogenic strain to inoculate cabbage, broccoli, Japanese radish, Chinese cabbage, tomato, and oat. *Pcal* wild type (WT) and Δ*cmaA* were grown on King’s B (KB; [54]) medium at 28 °C NU19 and NB35 were grown on KB medium containing kanamycin (10 µg/mL) (Km) (Supplementary Table S1). Before *Pcal* inoculation, bacteria were suspended in sterile distilled H_2_O, and the bacterial cell densities at 600 nm (OD_600_) were measured using a Biowave CO8000 Cell Density Meter (Funakoshi, Tokyo, Japan).

### 4.2. Bacterial In Vitro Growth Measurements

WT and NU19 were grown at 28 °C on Luria–Bertani (LB; [55]) medium. The bacterial suspensions were standardized to an OD_600_ of 0.01 with LB, and bacterial growth was measured at OD_600_ for 6, 9, 12, and 24 h.

### 4.3. Plant Materials

Plants used for *Pcal* virulence assays include cabbage (*Brassica oleracea* var. *capitate*) cv. Kinkei 201, broccoli (*Brassica oleracea* var. *italica*) cv. Midoribue, Japanese radish (*Raphanus sativus* var. *longipinnatus*) cv. Natsutsukasa, Chinese cabbage (*Brassica rapa* var. *pekinensis*) cv. Akimeki, tomato (*Solanum lycopersicum*) cv. Moneymaker, and oat (*Avena strigosa*) cv. Hayoat. All plants were grown from seed at 23–25 °C with a light intensity of 200 μEm^−2^s^−1^ and a 16 h light/8 h dark photoperiod. Seedlings were used for dip-inoculation assays around two weeks after germination.

### 4.4. Bacterial Inoculation

To assay for disease on cabbage, broccoli, Japanese radish, Chinese cabbage, tomato, and oat plants, dip inoculations were conducted by soaking seedlings in bacterial suspensions (5 × 10^7^ CFU/mL) containing 0.025% Silwet L-77 (OSI Specialities, Danbury, CT, USA). The seedlings were then incubated in growth chambers at 85–95% RH for the first 24 h, then at 80–85% RH for the rest of the experimental period. Disease symptoms were photographed at 5 days postinoculation (dpi) for all plants. To assess bacterial growth in all plants, the internal bacterial populations were measured after dip inoculation. Inoculated seedlings were collected, and two inoculated leaves were measured. The leaves were surface-sterilized with 10% H_2_O_2_ for 3 min. After washing with sterile distilled water three times, the leaves were homogenized in sterile distilled water, and diluted samples were plated onto solid KB agar medium. Two or three days after dilution sample plating, the bacterial colony-forming units (CFUs) were counted and normalized as CFU per gram, using the total leaf weight. The bacterial populations at 0 dpi were estimated using leaves harvested at 1 hpi without surface sterilization. The bacterial populations were evaluated in at least three independent experiments.

Cabbage was syringe-inoculated with *Pcal* WT, Δ*cmaA*, and NB35 (5 × 10^7^ CFU/mL) with a 1 mL blunt syringe. The plants were then incubated at 70–80% RH for the rest of the experimental period. To assess bacterial growth in cabbage, the internal bacterial population was measured at 6 hpi. Leaf disks were harvested using a 3.5 mm-diameter cork-borer from syringe-infiltrated zones. The bacterial populations were evaluated in at least three independent experiments.

### 4.5. Monitoring Gene Expression in Planta

To analyze plant gene expression profiles during infection, we syringe-inoculated cabbage plants with *Pcal* WT (5 × 10^5^ CFU/mL), and sampled at 24 and 48 hpi. To compare gene expression profiles during infection, cabbage plants were syringe-inoculated with *Pcal* WT, Δ*cmaA*, and NB35 (5 × 10^7^ CFU/mL), and sampled at 6 hpi, where the bacterial populations of even the virulence pathogen WT had not yet significantly increased. The total RNAs, including plant and bacterial RNAs, were extracted from infected leaves and purified. Total RNA extraction and real-time quantitative RT-PCR (RT-qPCR) were performed as described previously [56]. Two micrograms of total RNA were treated with gDNA Remover (Toyobo, Osaka, Japan) to eliminate genomic DNA, and the DNase-treated RNA was reverse-transcribed using the ReverTra Ace qPCR RT Master Mix (Toyobo). The cDNA (1:10) was then used for RT-qPCR using the primers shown in Table S2 with THUNDERBIRD SYBR qPCR Mix (Toyobo) on a Thermal Cycler Dice Real-Time System (Takara Bio, Kusatsu, Japan). Cabbage *UBIQUITIN EXTENSION PROTEIN* 1 (*BoUBQ1*) was used as an internal control to normalize gene expression. The reagent blank (no-template) controls were used to detect contamination. The expression profiles were evaluated in at least six independent samples.

### 4.6. Brassinin Quantification by RP-LC-ESI-MS/MS

*Pcal* WT bacterial suspension (5 × 10^5^ CFU/mL), or water (mock) were infiltrated into three-week-old cabbage. Twenty leaf discs (3.5 mm diameter) from four cabbage leaves were collected 48 hpi, the weight was measured, and samples were frozen in liquid nitrogen and stored at −80 °C. Samples were extracted with 300 μL of 80% methanol.

Brassinin was measured by using the multiple reaction monitoring (MRM) mode on the LC-ESI-MS/MS (LCMS-8045; Shimadzu, Kyoto, Japan) under the following conditions: capillary voltage, 4.5 kV; desolvation line, 300 °C; heat block, 500 °C; nebulizer nitrogen gas 3 L/min; drying gas, 10 L/min. Ion-source polarity was set in the negative-ion mode. The separation was performed with the LC system equipped with a 150 × 2.1 mm ACQUITY UPLC CSH C18 Column (Waters Corp., Milford, MA, USA) with a particle and pore size of 1.7 μm and 130Å, respectively. The initial mobile phase was solvent A: solvent B = 95:5 (solvent A, 0.025% formic acid; solvent B, acetonitrile (LC/MS Grade, Merck KGaA, Darmstadt, Germany) and maintained for 4 min. The solvent B concentration was increased to 50% for 11 min and then maintained at that ratio for another 5 min. The column was re-equilibrated for 3 min. The 0.4 mL min^−1^ flow rate and the 40 °C column temperature were maintained throughout the analysis. The MRM-transition m/z 235→58 was used as a precursor and as productions, respectively. The dwell time, Q1 pre-bias, collision energy, and Q3 pre-bias were set at 100 ms, 26 V, 7 eV, 21 V, respectively. The brassinin ion peak was detected at the retention of 15.3 min, and the fragment ion peak area of *m*/*z* = 58 was used for the quantification.

### 4.7. Antimicrobial-Activity Assay

To analyze brassinin antimicrobial activity against bacteria, the *Pcal* suspension was standardized to an OD_600_ of 0.01 in LB and coincubated with or without 200 µM brassinin (Merck KGaA). After 24 h, bacterial growth was measured at OD_600_.

### 4.8. Inhibition Assay

To analyze WT and NU19 susceptibility to plant-derived antimicrobials, WT and NU19 were grown at 28 °C on KB medium. The bacterial suspensions were standardized to an OD_600_ of 0.01 with KB, and after 6 h incubation, 200 µM antimicrobials, including brassinin (Merck KGaA), sulforaphane (Funakoshi), camalexin (Merck KGaA), daidzein (INDOFINE Chemical Company, Hillsborough, NJ, USA), genistein (Tokyo Chemical Industry, Tokyo, Japan), indole (Tokyo Chemical Industry), and phloretin (Funakoshi), was added to each sample. Bacterial growth was measured at OD_600_ after 24 h incubation.

### 4.9. Drug-Susceptibility Tests

The minimum inhibitory concentrations (MICs) of antibiotics for WT and NU19 were determined via cell growth in 2-fold dilutions of test compounds, including spectinomycin, streptomycin, nalidixic acid, cefotaxime, tetracycline, ampicillin, and carbenicillin (Merck KGaA), in 96-well plates containing KB medium to reach a total volume of 100 µL per well. The bacterial suspensions were standardized to an OD_600_ of 0.01 with KB, and bacterial growth was examined by visual inspection after 24 h of static incubation.

### 4.10. Statistical Analysis

All data are expressed as the mean with SE. All statistical analyses were performed using EZR (Saitama Medical Centre, Jichi Medical University, Saitama, Japan; [57]), a graphical user interface for R (version 3.6.3; R Foundation for statistical Computing, Vienna, Austria). Tukey’s honestly significant difference (HSD) test was used to analyze gene expression profiles. Differences of *p* < 0.05 were considered statistically significant.

## Figures and Tables

**Table 1:** Antimicrobial susceptibility of *Pcal* KB211 WT and NU19.

	MIC (µg/mL) in KB Medium						
Strain	Sp	Sm	Nal	Cef	Tet	Amp	Car
WT	8	4	4	16	1	32	>1000
NU19	4	2	4	16	1	32	>1000

Sp, Spectinomycin; Sm, Streptomycin; Nal, Nalidixic acid; Cef, Cefotaxime; Tet, Tetracycline; Amp, Ampicillin; Car, Carbenicillin.

## Data Availability

The data presented in this study are openly available in Supplementary Materials here.

## Supplementary Materials

The following supporting information can be downloaded at: https://www.mdpi.com/article/10.3390/plants11131742/s1, Table S1: Bacterial strains and plasmids used in this study; Table S2: Primer sets used in this study; Figure S1: *Pseudomonas cannabina* pv. *alisalensis* KB211 WT and NU19 growth in KB medium; Figure S2: Expression profiles of *CYP83A1* (A), *FMOG-OX2* (B), *FMOG-OX5* (C), *CYP81F2* (D), *CYP81F4* (E) were determined 24 and 48 h after inoculation with 5 × 10^5^ CFU/mL of WT or mock water-inoculated control, using real-time quantitative reverse transcription PCR with gene-specific primer sets; Figure S3: Disease symptoms on broccoli (A), Japanese radish (B), Chinese cabbage (C), tomato (D), and oat (E) dip-inoculated with *Pseudomonas cannabina* pv. *alisalensis* KB211 WT and NU19; Figure S4: Bacterial populations in cabbage syringe-inoculated with *Pseudomonas cannabina* pv. *alisalensis* KB211 WT, Δ*cmaA*, and NB35 (TTSS mutant); Spreadsheets: The data presented in this study.
