# Supplementary material for: Pseudomonas cannabina pv. alisalensis Virulence Factors Are Involved in Resistance to Plant-Derived Antimicrobials during Infection
Source: Plants (Basel). 2022 Jun 30;11(13):1742. doi: 10.3390/plants11131742 (PMC9269351; doi:10.3390/plants11131742)
Supplement: Supplementary file 1 [file plants-11-01742-s001.zip › Figure S1.pdf]

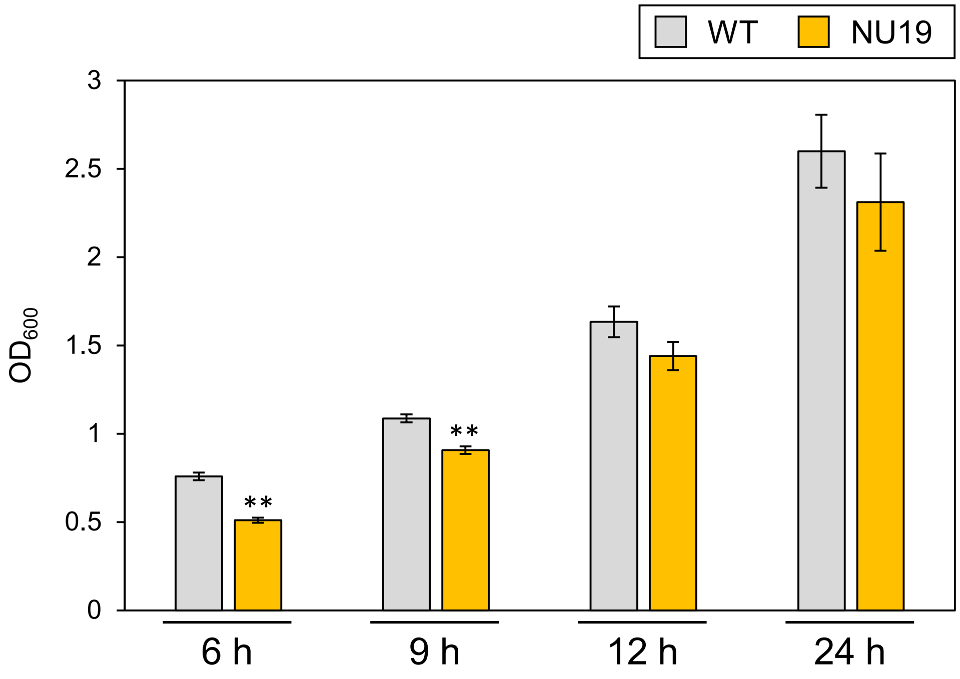

Figure S1. *Pseudomonas cannabina* pv. *alisalensis* KB211 WT and NU19 growth in KB medium. These strains were adjusted to an OD_{600} of 0.01 in KB medium and incubated with shaking at 28°C. Bacterial growth was quantified at 6, 9, 12, and 24 h. Vertical bars indicate the standard error for three biological replicates. Asterisks indicate a significant difference from the water treatment control in a t-test (**$p < 0.01$).
