# Supplementary material for: Pseudomonas cannabina pv. alisalensis Virulence Factors Are Involved in Resistance to Plant-Derived Antimicrobials during Infection
Source: Plants (Basel). 2022 Jun 30;11(13):1742. doi: 10.3390/plants11131742 (PMC9269351; doi:10.3390/plants11131742)
Supplement: Supplementary file 1 [file plants-11-01742-s001.zip › Figure S2.pdf]

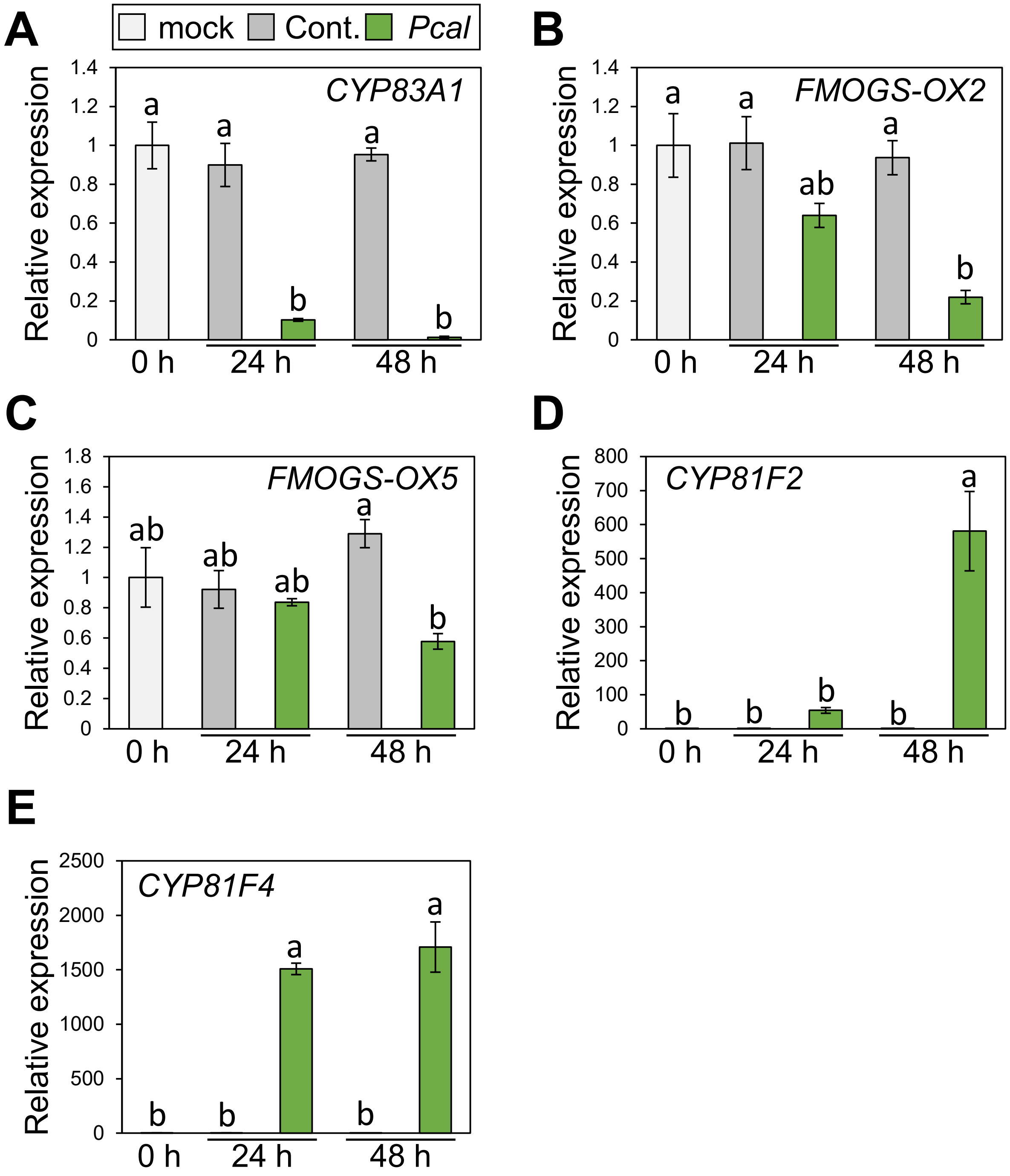

Figure S2. Expression profiles of *CYP83A1* (A), *FMOG-OX2* (B), *FMOG-OX5* (C), *CYP81F2* (D), *CYP81F4* (E) were determined 24 and 48 h after inoculation with 5 x 10^5 CFU/ml of WT or mock water-inoculated control, using real-time quantitative reverse transcription PCR with gene-specific primer sets. Expression in cabbage was normalized using *BoUBQ1*. Vertical bars indicate the standard error for three biological replicates. Different letters indicate a significant difference among treatments based on a Tukey's honestly significant different test (p <0.05).
