# Supplementary material for: Pseudomonas cannabina pv. alisalensis Virulence Factors Are Involved in Resistance to Plant-Derived Antimicrobials during Infection
Source: Plants (Basel). 2022 Jun 30;11(13):1742. doi: 10.3390/plants11131742 (PMC9269351; doi:10.3390/plants11131742)
Supplement: Supplementary file 1 [file plants-11-01742-s001.zip › Figure S3.pdf]

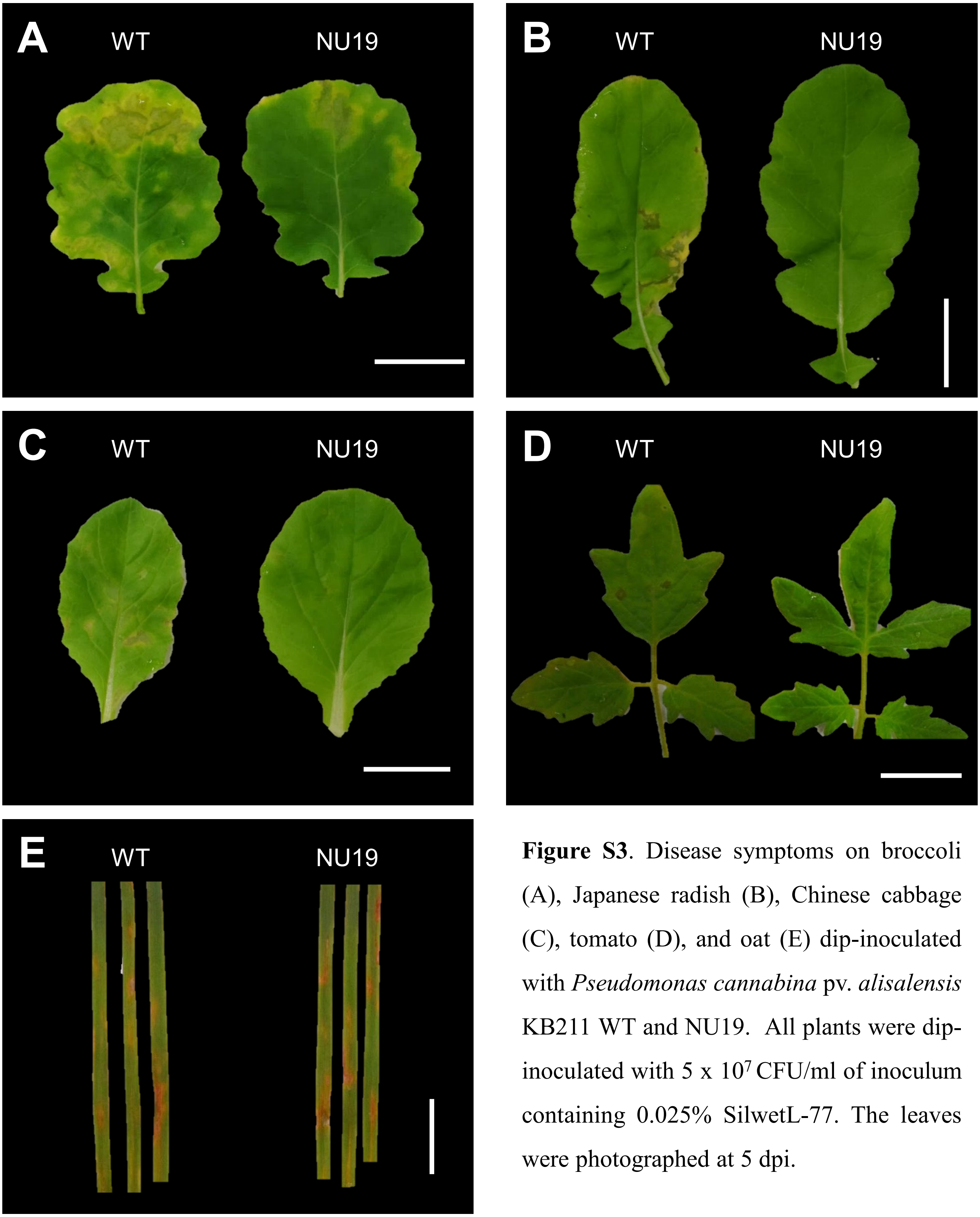

Figure S3. Disease symptoms on broccoli (A), Japanese radish (B), Chinese cabbage (C), tomato (D), and oat (E) dip-inoculated with *Pseudomonas cannabina* pv. *alisalensis* KB211 WT and NU19. All plants were dip-inoculated with 5 x 10^7 CFU/ml of inoculum containing 0.025% SilwetL-77. The leaves were photographed at 5 dpi.
