# Supplementary material for: Pseudomonas cannabina pv. alisalensis Virulence Factors Are Involved in Resistance to Plant-Derived Antimicrobials during Infection
Source: Plants (Basel). 2022 Jun 30;11(13):1742. doi: 10.3390/plants11131742 (PMC9269351; doi:10.3390/plants11131742)
Supplement: Supplementary file 1 [file plants-11-01742-s001.zip › Figure S4.pdf]

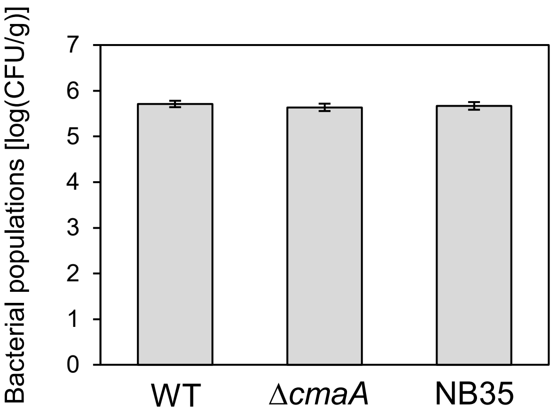

Figure S4. Bacterial populations in cabbage syringe-inoculated with *Pseudomonas cannabina* pv. *alisalensis* KB211 WT, Δ*cmaA*, and NB35 (TTSS mutant). Cabbage plants were syringe-inoculated with 5 x 10^7 CFU/ml of inoculum. The bacterial populations in the plant were evaluated at 6 hpi. Vertical bars indicate the standard error for at least three independent experiments.
