# Supplementary material for: Pseudomonas cannabina pv. alisalensis Virulence Factors Are Involved in Resistance to Plant-Derived Antimicrobials during Infection
Source: Plants (Basel). 2022 Jun 30;11(13):1742. doi: 10.3390/plants11131742 (PMC9269351; doi:10.3390/plants11131742)
Supplement: Supplementary file 1 [file plants-11-01742-s001.zip › Table S1.pdf]

Table S1. Bacterial strains and plasmids used in this study

Bacterial strain or plasmid	Relevant characteristics	Reference or source
P. cannabina pv. *alisalensis*		
Isolate KB211	Wild type, Rif ^{r}	Nagano vegetable and ornamental crops experiment station
P. cannanbina pv. *alisalensis* Tn*5* mutants		
NU19	Tn*5* mutant containing transposon in gene encoding resistance-nodulation-cell division (RND) transporter, Rif ^{r}, Km ^{r}, Cm ^{r}	Sakata et al. (2019)
NB35	Tn*5* mutant containing transposon in gene encoding type III secretion protein HrcQb, Rif ^{r}, Km ^{r}, Cm ^{r}	Sakata et al. (2019)
Δ*cmaA*	*Pcal* KB211 Δ*cmaA*, Rif ^{r}	Sakata et al. (2021)

Cm ^{r} chloramphenicol resistance, *Km* ^{r} kanamycin resistance, *Rif* ^{r} rifampicin resistance
