# Supplementary material for: Pseudomonas cannabina pv. alisalensis Virulence Factors Are Involved in Resistance to Plant-Derived Antimicrobials during Infection
Source: Plants (Basel). 2022 Jun 30;11(13):1742. doi: 10.3390/plants11131742 (PMC9269351; doi:10.3390/plants11131742)
Supplement: Supplementary file 1 [file plants-11-01742-s001.zip › Table S2.pdf]

Table S2. Primer sets used in this study

Gene	Primer name	Primer sequence	Reference
BoUBQ1	BoUBQ1-FW	GTCAAGGCCAAGATCCAAGA	Ishiga et al. 2020
BoUBQ1	BoUBQ1-RV	GGATGTTGTAGTCAGCCAGAG	Ishiga et al. 2020
BoCYP83B1	*BoCYP83B1*-FW	CTTTCGTCTCTCCAAGCTCTAC	This study
BoCYP83B1	*BoCYP83B1*-RV	TTGAGGAGTTCCTTGGCTAAC	This study
BoBABG.a	*BoBABG.a*-FW	GTTCCGGTTCAAGTGGTATCT	This study
BoBABG.a	*BoBABG.a*-RV	GTTTCCTCCTCCTCTTCTTCAC	This study
BoBABG.b	*BoBABG.b*-FW	GGACAGAGTACCACAAGAAACA	This study
BoBABG.b	*BoBABG.b*-RV	GTCTAGTAAGGACCAGGCAAAG	This study
BoDTCMT	*BoDTCMT*-FW	TTGAGGACAGTGGGTGTTTC	This study
BoDTCMT	*BoDTCMT*-RV	TGTGTGAGGTTACCAAGAACTC	This study
BoCYP83A1	*BoCYP83A1*-FW	ACTGAAGACGACGTGAAGAAC	This study
BoCYP83A1	*BoCYP83A1*-RV	ATGCAAGCACGAGGGATAAG	This study
BoFMOGS-OX2	*BoFMOGS-OX2*-FW	GGATTAATAGCGGCCAGAGAG	This study
BoFMOGS-OX2	*BoFMOGS-OX2*-RV	GCGGGTCGGATTCAGATTTA	This study
BoFMOGS-OX5	*BoFMOGS-OX5*-FW	GACTCTGTGGTCGTGTGTAATG	This study
BoFMOGS-OX5	*BoFMOGS-OX5*-RV	CAGGAACTCTGTAGTTGTGACTATG	This study
BoCYP81F2	*BoCYP81F2*-FW	GCATGAGGCCTTTGATGATGAC	This study
BoCYP81F2	*BoCYP81F2*-RV	GAGCATATACGGCGGAGATTAC	This study
BoCYP81F4	*BoCYP81F4*-FW	CGTCTGCTTGATGAGTGTAGAGG	This study
BoCYP81F4	*BoCYP81F4*-RV	CTGGGTCTTGTTGTTGCAAAG	This study
